# Re-evaluation of the pathogenic roles of nonstructural protein 1 and its antibodies during dengue virus infection

**DOI:** 10.1186/1423-0127-20-42

**Published:** 2013-06-27

**Authors:** Yung-Chun Chuang, Shu-Ying Wang, Yee-Shin Lin, Hong-Ru Chen, Trai-Ming Yeh

**Affiliations:** 1 Center of Infectious Disease and Signaling Research, Medical College, National Cheng Kung University, Tainan, Taiwan; 2 Department of Microbiology and Immunology, Medical College, National Cheng Kung University, Tainan, Taiwan; 3 Institute of Basic Medical Sciences, Medical College, National Cheng Kung University, Tainan, Taiwan; 4 Department of Medical Laboratory Science and Biotechnology, Medical College, National Cheng Kung University, Tainan, Taiwan

**Keywords:** Endothelium, Permeability, Hemorrhage, Pathogenesis

## Abstract

Dengue virus (DENV) infection can cause life-threatening dengue hemorrhagic fever (DHF) and dengue shock syndrome (DSS). Vascular leakage and abnormal hemorrhage are the two major pathogenic changes found in these patients. From previous studies, it is known that both antibodies and cytokines induced in response to DENV infection are involved in the immunopathogenesis of DHF/DSS. However, the role of viral factors during DENV infection remains unclear. Nonstructural protein 1 (NS1), which is secreted in the sera of patients, is a useful diagnostic marker for acute DENV infection. Nevertheless, the roles of NS1 and its antibodies in the pathogenesis of DHF/DSS are unclear. The focus of this review is to evaluate the possible contributions of NS1 and the antibodies it induces to vascular leakage and abnormal hemorrhage during DENV infection, which may provide clues to better understanding the pathogenesis of DHF/DSS.

## Review

### Introduction

Dengue virus (DENV) belongs to the genus flavivirus and is a positive-stranded enveloped RNA virus. The RNA is approximately 10.1 Kb and is translated into three structural proteins: core protein (C), membrane-associated protein (M) produced as a precursor protein (prM) and envelope protein (E). Additionally, there are 7 nonstructural proteins (NS), including NS1, NS2a, NS2b, NS3, NS4a, NS4b and NS5. Based on the antigenic differences of the E protein, DENV can be subgrouped into four different serotypes: DENV 1, 2, 3, and 4 [1-3].

DENV infection is transmitted by *Aedes* mosquitoes. It is prevalent in tropic and sub-tropic areas where the vector resides. It has been estimated that greater than 2.5 billion people live in endemic areas, and the number of individuals infected by DENV is thought to exceed 50 million globally per year [4,5]. Most DENV infections cause flu-like symptoms, such as fever, headache, muscle and bone pain. This infection is referred to as dengue fever (DF), and it naturally resolves in several days. However, in some patients, severe dengue hemorrhagic fever/dengue shock syndrome (DHF/DSS) may occur. This is correlated with high viremia, secondary dengue virus infection, and DENV type 2 [6-8]. The characteristic features of DHF/DSS include vascular (plasma) leakage, thrombocytopenia, and coagulopathy. Due to a lack of knowledge regarding the process leading to DHF/DSS, only supportive treatment is currently available [9]. In addition, vector control is the only method of prevention, as there is no effective vaccine currently available for DENV [10]. Therefore, further study of the host and viral factors of dengue pathogenesis is crucial for developing effective vaccines and drugs to prevent the occurrence of DHF/DSS [11,12].

**Figure 1:**
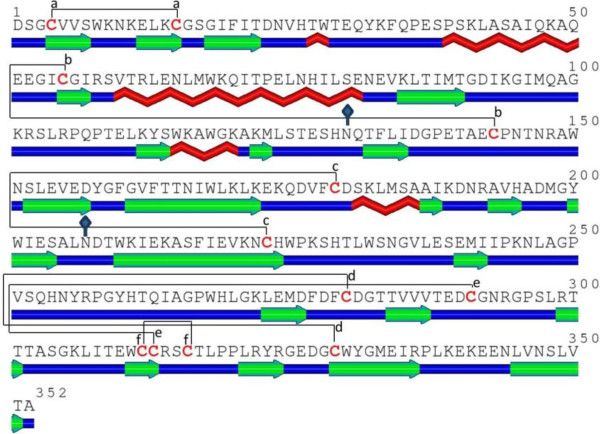
**Amino acid sequence and secondary structure of DENV type 2 NS1 protein predicted by SABLE**[22]**.** The elements are color coded as follows: red, α-helix; green, β-sheet; blue, coil. Linkages of six disulfide bonds (**a**-**f**) are represented with solid lines. Two potential N-glycosylation sites are represented with solid diamonds.

Flavivirus NS1 is a relatively conserved glycoprotein with a molecular weight of 46–55 kDa, depending on its glycosylation status, which exists in different forms at different cellular locations [13]. Immature NS1 exists as a monomer in the endoplasmic reticulum, and it is processed into a stable homodimer that can be covalently linked to the surface membrane via a glycosyl-phosphatidylinositol anchor [14]. Mature DENV NS1 contains 352 amino acid residues with two N-linked glycosylation sites at residues 130 and 207. There are 12 cysteine residues in DENV NS1 that are absolutely conserved among all flavivirus NS1 proteins, indicating the importance of disulfide bonds in the structure and function of NS1 (Figure 1) [15]. Unlike other nonstructural proteins, DENV NS1 can also be secreted as a soluble hexamer, which forms a lipoprotein particle with an open-barrel protein shell and a prominent central channel rich in lipids [16,17]. NS1 antigen circulates in dengue patients from the first day after the onset of fever up to day 9, when the clinical phase of the disease is over [18]. The serum levels of NS1 are estimated to range from 0.01 to 50 μg/ml and early concentrations of NS1 in blood are positively associated with disease severity [19]. Therefore, DENV NS1 antigen detection has been successfully used for the early diagnosis of DENV infection [20,21].

Despite the many gaps in our knowledge of the structure and function of flavivirus NS1, it is known that intracellular NS1 co-localizes with dsRNA and other components of replication complexes and plays an essential cofactor role in virus replication [13,23,24]. Conversely, secreted NS1 has been shown to bind a number of different complement pathway components [25]. Complement activation mediated by DENV NS1, which leads to local and systemic generation of anaphylatoxins and the membrane attack complex, may contribute to the pathogenesis of the vascular leakage that occurs in DHF/DSS patients [26]. In fact, reduction in the levels of complement components have been described in DHF/DSS patients, suggesting that complement activation may have a role in the pathogenesis of severe disease [27]. In addition, both secreted and membrane-associated DENV NS1 are highly immunogenic, and the antibodies they elicit can cross-react with human endothelial cells and platelets [28,29]. Therefore, both NS1 and its antibodies may play pivotal roles in the pathogenesis of DHF/DSS.

### Pathogenesis of vascular leakage in DHF/DSS

The most prominent feature of DHF/DSS and the best indicator of disease severity is plasma leakage [30,31]. Plasma leakage is caused by an increase in the capillary permeability, and it manifests as any combination of hemoconcentration, plural effusion, or ascites. It usually becomes evident on days 3–7 of illness, at which point dengue fever resolves (defervescence), the viral titer drops, and anti-DENV antibodies gradually increase. However, the underlying pathophysiological mechanisms of plasma leakage in DHF/DSS are not fully understood.

**Figure 2:**
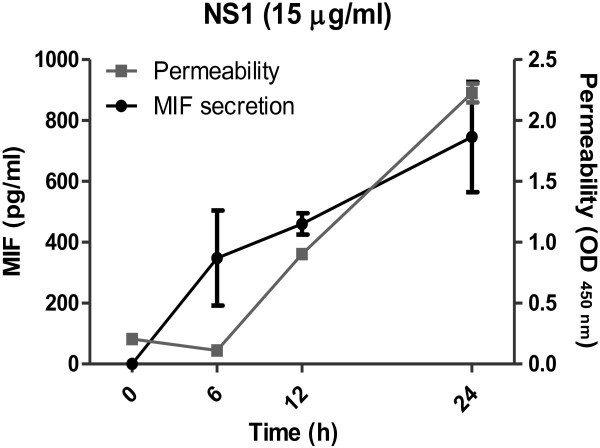
**Permeability changes and MIF secretion of human endothelial HMEC-1 cells induced by DENV rNS1.** DENV rNS1 (15 μg/ml), prepared as previously described [32], was incubated with HMEC-1 cells. The secretion of MIF in the medium was measured by ELISA at different time points as indicated. The permeability of HMEC-1 cells was determined by a transwell assay as previously described [33]. Each point represents the mean ± SEM in duplicate.

It is known that the vascular endothelium plays important roles in the regulation of tissue fluid homeostasis and transmigration of leukocytes [34,35]. Endothelial cells and their associated structures, such as the glycocalyx and basement membrane, form the primary semipermeable barrier, which is tightly regulated in the resting state. During inflammation, the tight junction between adjacent endothelial cells and the surface glycocalyx of the endothelium are acutely or permanently modified as a part of the immune response [36,37]. Thus, vascular permeability is increased, followed by leukocyte adherence and coagulation activation. Although there are differences in the endothelial cell architecture in capillaries of different organs, the phenotypic changes associated with endothelial hyperpermeability are similar.

Evidence for endothelial cell activation during DENV infection has been reported [38,39]. Electron microscopy studies have shown vacuolation of the cytoplasm of endothelial cells and gap formations in the endothelial junctions of skin biopsies of DHF patients. Although DENV has been shown to infect endothelial cells *in vitro*[40], histological studies on viral antigens in tissue specimens suggest that direct infection of endothelial cells by DENV occurs only occasionally [41,42]. Nevertheless, many pro-inflammatory cytokines, such as tumor necrosis factor-α (TNF-α), macrophage migration inhibitory factor (MIF), monocyte chemotactic protein-1 (MCP-1), interleukin-8 (IL-8), and high mobility group box-1 (HMGB-1) are increased during DENV infection, which may contribute to vascular hyperpermeability through the disruption of the tight junction and glycocalyx degradation [33,43-46]. Because pathogenic changes of plasma leakage are reversible, it is generally believed that physical damage is not involved. Instead, soluble mediators, such as cytokines produced during the acute phase of infection, likely play an important role in the pathogenesis of DHF/DSS [47,48]. However, it is difficult to reconcile the specific vascular leakage in DENV infection with cytokines or similar bio-active mediators because they are also generated during other infections that do not lead to vascular leakage. Therefore, a more complex interaction between host and virus factors has yet to be characterized in the pathogenesis of vascular leakage in DHF/DSS.

### Pathogenesis of thrombocytopenia and coagulopathy in DHF/DSS

In addition to vascular leakage, almost all DHF patients have abnormal hemostasis, which is evidenced by marked thrombocytopenia (platelet count less than 100,000/μl) [49]. In fact, thrombocytopenia is one of the most consistent clinical features of severe dengue infection [50]. This occurs as a result of both decrease of platelet production due to DENV-induced bone marrow suppression and increase of platelet destruction in blood circulation [51,52]. Immune complexes containing dengue antigen have been reported on platelet surfaces and may be one of the mechanisms underlying increased platelet destruction [53,54]. Subsequent studies have shown that autoantibodies that can cross-react with platelets are induced in DENV patients and NS1 immunized mice [55,56]. These anti-platelet antibodies may represent another possible mechanism by which platelet consumption is increased during DENV infection.

In addition to thrombocytopenia, an association between coagulation and fibrinolysis activation and clinical outcome is also conceivable [57,58]. Prolonged prothrombin time and activated partial thromboplastin time (APTT), reduced fibrinogen level, and increased fibrinogen degradation products are more common in DHF than DF [59,60]. Normally, hemostasis is tightly controlled to prevent overt bleeding or thrombosis. Thus far, it remains unclear how hemorrhage is induced during DENV infection. However, autoantibodies and cytokines induced by DENV infection, hemostatic molecules expressed on DENV-infected cells, and DENV viral proteins may all contribute to the defect of hemostasis during DENV infection [61]. The combination of these viral and host factors may tilt the balance of coagulation and fibrinolysis toward bleeding in dengue patients.

### Possible pathogenic effects of anti-NS1 cross-reactive antibodies during DENV infection

Several hypotheses have been proposed to explain the pathogenesis of DHF/DSSincluding antibody-dependent enhancement (ADE) [52]. The ADE hypothesis was proposed to explain why DHF/DSS occurs more commonly in secondary-infected patients with a different serotype of DENV. According to ADE, antibodies against DENV structural proteins, such as E protein or prM, which are generated from previous infections, cannot efficiently neutralize subsequent DENV infections of a different serotype. Rather, these antibodies could bind to DENV andenhance its infection to Fcγ receptor-positive cells such as macrophage [62]. The ADE hypothesis leads us to realize the potential pathogenic roles of antibody in dengue pathogenesis and explains why passive and actively acquired dengue antibodies may result in enhance infections. However, the pathogenic mechanisms of vascular leakage and hemorrhage in DHF/DSS patients are still unclear. Recently, anti-NS1 antibodies that can cross-react with different coagulation-related molecules and cells, such as human plasminogen, thrombin, platelets and endothelial cells, have been reported [63-67]. The titers of these endothelial cells and platelets cross-reactive anti-NS1 antibodies were higher in the acute phaseof DHF/DSS patientsthan those in DF patients. In addition, the titers of these autoantibodies are decreased in the sera collected in the convalescent phase, even though the total anti-NS1 antibody levels continually remain high [55,68]. This may explain why there is no subsequent autoimmune disease in DHF patients once they are recovered. These anti-NS1 autoantibodies can lead to thrombocytopenia *in vivo*[56,69] and nitric oxide-mediated apoptosis of endothelial cells in vitro[64]. Because there is sequence homology between DENV NS1 and proteins on endothelial cells and platelets, it is possible that these autoantibodies are induced by NS1 through molecular mimicry [70]. Therefore, autoantibodies induced by NS1 may contribute to thrombocytopenia, coagulopathy and vascular leakage in DHF/DSS. However, these symptoms in DHF/DSSpatients usually occur within the first week of fever onset when antibodies are still underdeveloped [7,31,71]. Therefore, we think the pathogenic roles of secreted NS1 cannot be neglected, especially in the early stage of DENV infection.

### Possible pathogenic effects of NS1 during DENV infection

NS1, which is secreted early during DENV infection, can bind to heparan sulfate on the surface of a wide variety of cells, including epithelial cells, fibroblasts, hepatocytes and some endothelial cells [72]. The binding of NS1 to the surface of endothelial cells can induce complement activation, which may contribute to the pathogenesis of vascular leakage that occurs in patients with DHF/DSS [26]. Secreted NS1 can also be endocytosed by hepatocytes, which may enhance DENV infection [73]. Binding of anti-NS1 antibodies to membrane-anchored NS1 can also induce signal transduction, leading to protein tyrosine phosphorylation that might affect DENV replication within infected cells [14]. Conversely, NS1 interacting with complement protein C4 and C4b-binding protein, which can promote C4 degradation, may in turn protect DENV from complement-dependent lysis [74,75]. Therefore, NS1 is a viral factor that can enhance both DENV replication and immune evasion.

**Figure 3:**
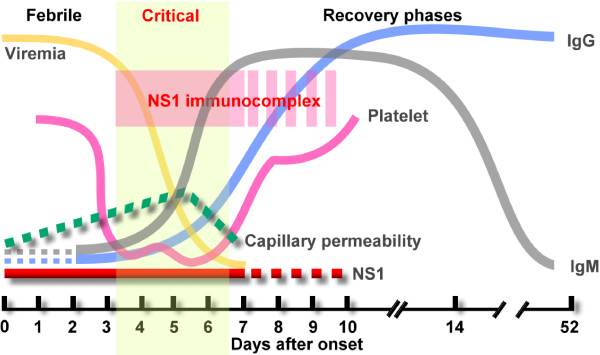
**Viremia, NS1 antigen and antibody responses during DENV infection.** A schematic demonstration of the relationship between vascular leakage, thrombocytopenia, the kinetics of DENV viremia, the detection of secreted NS1, and titers of anti-DENV antibodies in the sera of dengue patients during febrile, critical, and recovery phases of the disease.

Recently, we have demonstrated that DENV NS1 can bind to prothrombin and inhibit its activation, which may prolong APTT in dengue patients [32]. This may explain why APTT abnormality occurs within the first week of fever onset when the antibody response is still weak. In addition, because the vascular leakage in dengue patients is directly correlated with APTT levels, NS1 may also contribute to plasma leakage by mechanisms that do not involve antibodies. In our preliminary study using recombinant DENV NS1 (rNS1) to stimulate the human endothelial cell line HMEC-1, we found that rNS1 stimulation of HMEC-1 cells increased cell permeability in a time-dependent manner, which was positively correlated with the secretion of MIF in the culture medium (Figure 2). Even though further studies using native forms of NS1 are required to confirm the effect of NS1 on the permeability of endothelial cells, it is possible that NS1 may induce cytokine production, such as MIF, to cause vascular leakage in the early stages of DENV infection. NS1 can be detected early during the disease before antibodies are induced, and the levels of NS1 rapidly decrease in the circulation once anti-NS1 antibodies increase [18]. Therefore, peak NS1 is only observed early during the acute phase of infection. As a consequence, the high levels of NS1 do not coincide with the onset of severe disease [19]. In addition, the magnitude of NS1 secretion does not correlate with severe pathology in the mouse model [76]. Thus, we propose that the pathogenic effect of DENV infection on vascular permeability may reach the peak when anti-NS1 antibodies are induced and NS1 immunocomplexes are formed. Besides to the pathogenic effects of NS1 and its antibodies which were mentioned above, complement activation by NS1 immunocomplexesmay further contribute to the development of thrombocytopenia and vascular leakage during the critical phase of DHF/DSS (Figure 3).

## Conclusions

**Figure 4:**
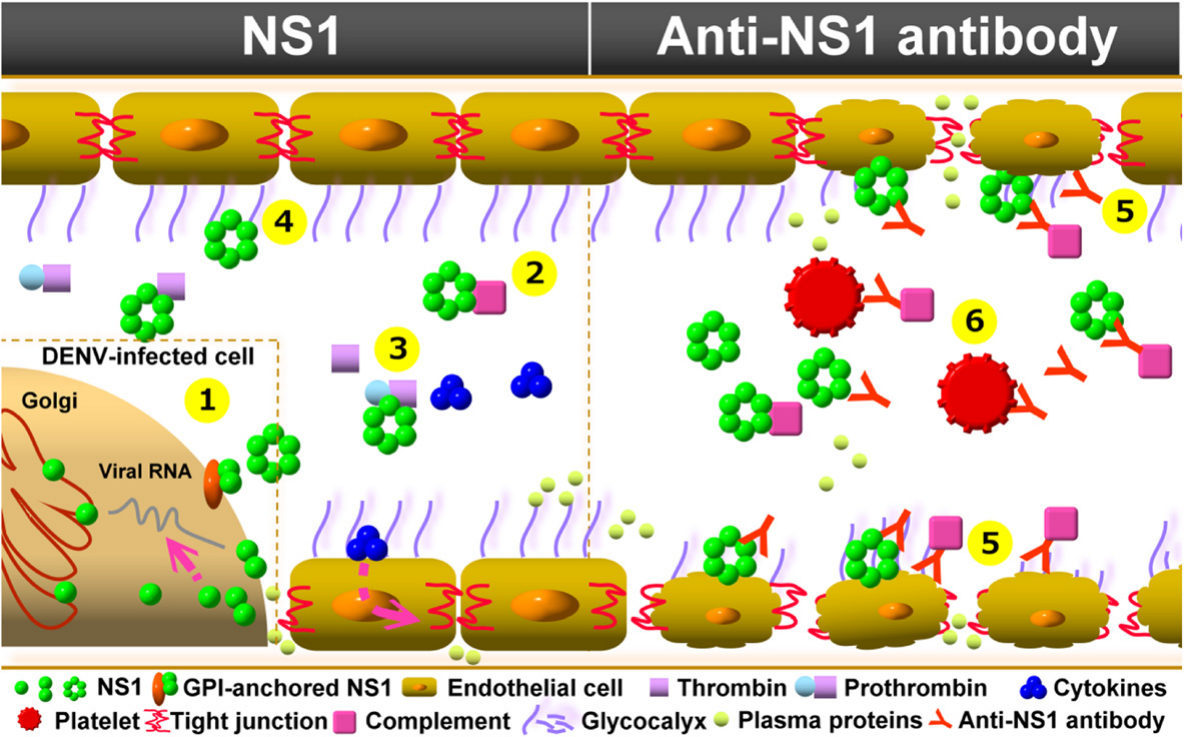
**Potential pathogenic roles of NS1 and its antibodies in DENV infection.** During DENV infection, NS1 can exist in monomeric, dimeric, or hexameric form. In the left panel, (1) NS1 is involved in DENV replication within cells [13,23,24]. The NS1 dimer can be anchored at the cell membrane, which may be involved in signaling transduction [14]. (2) NS1 can bind to C4 protein and promotes its degradation [74]. (3) NS1 can also bind to prothrombin and inhibit its activation [32]. (4) The binding of NS1 to cells may enhance endocytosis and cytokine production, which may enhance DENV infection and increase vascular permeability [73]. In the right panel, (5) anti-NS1 antibodies that cross-react with endothelial cells may induce endothelial damage [64]. (6) Anti-platelet antibodies elicited by NS1 may inhibit platelet aggregation and cause thrombocytopenia [56]. It is the integrated effects of NS1 and its antibodies, and the immunocomplexes they formed that may contribute to the development of thrombocytopenia, vascular leakage, and coagulopathy during the critical phase of DHF/DSS.

Not only is vascular leakage the hallmark of DHF/DSS, but there is evidence to suggest that damage during DENV infection may begin at endothelial surfaces. In clinical practice, plasma leakage-induced shock-related pathophysiological conditions are most often caused by bacterial infections such as sepsis or septic shock [77]. Regardless of the different causes, similar pathological changes are found in these patients. These are characterized by a systemic inflammatory response causing vascular hyperpermeability and leading to almost uncontrollable edema, coagulation changes and multi-organ failure. Bacterial cell wall components, such as lipopolysaccharide (LPS, or endotoxin), can induce septic shock. However, the viral factors in DHF/DSS remain unknown. Although it is premature to extrapolate from *in vitro* findings to human pathogenesis, it is possible that dengue NS1 may play a role similar to LPS for endotoxic shock patients to trigger the pathophysiological abnormalities in DHF/DSS [78]. However, it is the integrated effects of NS1, its antibodies, and the immunocomplexes they formed that may contribute to severe disease outcome (Figure 4). Further studies to investigate the pathogenic roles of NS1 and its antibodies, and of course the contribution of their interaction, immunocomplexes to the overall disease process may improve our current understanding of the pathogenesis of DHF/DSS and uncover new vaccine strategies and therapeutic approaches.

## Abbreviations

ADE: Antibody-dependent enhancement; APTT: Activated partial thromboplastin time; DENV: Dengue virus; DHF: Dengue hemorrhagic fever; DSS: Dengue shock syndrome; NS1: Nonstructural protein 1; LPS: Lipopolysaccharide.

## Competing interests

These authors declare no competing conflict.

## Authors’ contributions

YSL and TMY discussed and designed the concept. YCC, SYW, HRC, and TMY collected information and prepared the manuscript and figures. TMY wrote the manuscript. All the authors read and approved the final manuscript.
